# Exploring a novel therapeutic strategy: the interplay between gut microbiota and high-fat diet in the pathogenesis of metabolic disorders

**DOI:** 10.3389/fnut.2023.1291853

**Published:** 2023-12-15

**Authors:** Xiaokang Jia, Qiliang Chen, Huiwen Wu, Hongbo Liu, Chunying Jing, Aimin Gong, Yuanyuan Zhang

**Affiliations:** ^1^School of Traditional Chinese Medicine, Hainan Medical University, Haikou, Hainan, China; ^2^School of Basic Medicine, Guangzhou University of Chinese Medicine, Guangzhou, Guangdong, China; ^3^The Affiliated TCM Hospital of Guangzhou Medical University, Guangzhou, Guangdong, China

**Keywords:** gut microbiota, high-fat diet, metabolic disorders, pathogenesis, therapeutic strategies

## Abstract

In the past two decades, the rapid increase in the incidence of metabolic diseases, including obesity, diabetes, dyslipidemia, non-alcoholic fatty liver disease, hypertension, and hyperuricemia, has been attributed to high-fat diets (HFD) and decreased physical activity levels. Although the phenotypes and pathologies of these metabolic diseases vary, patients with these diseases exhibit disease-specific alterations in the composition and function of their gut microbiota. Studies in germ-free mice have shown that both HFD and gut microbiota can promote the development of metabolic diseases, and HFD can disrupt the balance of gut microbiota. Therefore, investigating the interaction between gut microbiota and HFD in the pathogenesis of metabolic diseases is crucial for identifying novel therapeutic strategies for these diseases. This review takes HFD as the starting point, providing a detailed analysis of the pivotal role of HFD in the development of metabolic disorders. It comprehensively elucidates the impact of HFD on the balance of intestinal microbiota, analyzes the mechanisms underlying gut microbiota dysbiosis leading to metabolic disruptions, and explores the associated genetic factors. Finally, the potential of targeting the gut microbiota as a means to address metabolic disturbances induced by HFD is discussed. In summary, this review offers theoretical support and proposes new research avenues for investigating the role of nutrition-related factors in the pathogenesis of metabolic disorders in the organism.

## 1 Introduction

With the improvement of living standards, metabolic diseases have been rampant worldwide. These diseases are closely related to high-fat diets (HFD) and pose a serious public health problem, leading to increased incidence and severity of chronic diseases such as obesity, diabetes, dyslipidemia, non-alcoholic fatty liver disease (NAFLD), hypertension, and hyperuricemia, as well as premature death (1, 2). By 2025, the global obesity rate is projected to reach around 20%, with severe obesity exceeding 6% (3). It is estimated that by 2035, the number of diabetes patients will increase to over 590 million (4). Dyslipidemia, hypertension, and hyperuricemia are also risk factors for cardiovascular diseases, and their prevalence is increasing year by year (5, 6). NAFLD is considered as the hepatic manifestation of metabolic syndrome associated with obesity, dyslipidemia, and hypertension, with an estimated prevalence of 25% in the general population (7). It is evident that the increasing prevalence of metabolic diseases poses a serious threat to the global human health.

A study found that after excluding factors such as geography, smoking, exercise, and energy intake, dietary patterns are closely associated with the risk of metabolic syndrome (8). Western-style diets, characterized by high fat intake, are associated with an increased risk of developing metabolic syndrome (8). On the other hand, another study found that the Mediterranean diet can modulate gut microbiota and its metabolites, improving cardiovascular metabolism (9). Further in-depth research has revealed that HFD can disrupt and alter gut microbiota, leading to dysfunction of the intestinal barrier and dysbiosis, which in turn contribute to the development of metabolic diseases (10–12). We hypothesize that gut microbiota may play a crucial role in the pathogenesis of metabolic diseases induced by HFD. This review aims to provide a brief overview of recent studies on the relationship between HFD, gut microbiota, and metabolic diseases, as well as discuss the potential mechanisms by which HFD alters gut microbiota composition.

## 2 HFD promotes the development of metabolic diseases

HFD significantly promotes the occurrence and development of metabolic diseases, primarily involving changes in various genes related to lipid metabolism, inflammation response, and insulin signaling pathways. In addition, HFD also leads to structural and functional changes in multiple organs, including the liver, intestines, and adipose tissue.

### 2.1 Obesity

Obesity is a diet-related chronic disease, and genetic predisposition to obesity can lead to increased hunger and loss of control over food intake. Recent research has found that a higher genetic propensity for body mass index (BMI) can be explained by the inhibition of satiety and hunger. Dietary restraint may play an important role in regulating susceptibility to high BMI, and a healthy diet can help prevent obesity (13).

Different dietary patterns have different effects on obesity. A sustainable diet refers to a dietary pattern that includes more plant-based foods and fewer animal-based foods. It is a diet that can be adhered to in the long term and is beneficial for both the environment and health. It has the potential protective effect against weight gain, overweight, and obesity risk (14, 15). HFD represents a significant and prominent feature within the traditional Western dietary pattern, which encompasses a substantial intake of saturated fats from meat, dairy products, and processed foods (8, 16–18). In Chinese adults, the traditional Chinese dietary pattern is significantly associated with a lower risk of overweight/obesity, while the Western dietary pattern is non-significantly associated with a higher risk of overweight/obesity (19). A meta-analysis showed that the Mediterranean diet and low-fat diet can reduce the mortality and non-fatal myocardial infarction risk in populations at increased cardiovascular risk (20). Another meta-analysis demonstrated that a low-fat diet can significantly reduce weight and improve cardiovascular risk factors after 6 months (21). A prospective cohort study found a positive association between a sulfur-microbiota diet and the risk of obesity and abdominal obesity, emphasizing the importance of avoiding a sulfur-microbiota diet in preventing obesity at all genetic risk levels (22). Investigating recent scholarly work, it is evident that feeding mice a high-energy diet with different fat/sugar ratios can induce obesity in mice (23). In a study of 151 obese women undergoing weight loss dietary intervention, the genotype of the apolipoprotein A5 (APOA5) gene, which is associated with obesity and cardiovascular metabolic risk, influenced the effectiveness of the dietary intervention. This finding provides a new research direction for personalized dietary interventions (24).

To investigate the mechanisms of HFD-induced obesity, a series of animal experiments have also been conducted. A mouse study showed that a HFD can induce changes in the morphology and function of hypothalamic microglia mitochondria, mediated by mitochondrial protein UCP2. These changes can activate microglia and neuroinflammation, affecting the regulation of appetite-controlling hypothalamic neurons and promoting obesity (25). Short-term HFD feeding can activate pro-opiomelanocortin (POMC) neurons in the arcuate nucleus (ARC) of mice, leading to excessive food intake. Removing PNOC-ARC in mice can promote the activation of POMC neurons during HFD feeding, reducing food intake and preventing HFD-induced obesity without affecting food intake and body weight during normal feeding (26). These findings demonstrate the close relationship between HFD and obesity, providing new insights for the prevention and treatment of obesity-related metabolic diseases.

### 2.2 Diabetes

Diet is closely related to diabetes. A healthy low-carbohydrate and low-fat diet is associated with a lower overall mortality rate in prediabetes, while an unhealthy low-carbohydrate and low-fat diet is associated with a higher overall mortality rate in prediabetes (27). Vegetable fat is more beneficial in preventing the occurrence of type 2 diabetes compared to animal fat (28). A study on overweight or obese individuals with prediabetes or moderately controlled type 2 diabetes found that the low-fat diet group could reduce blood glucose and glycated hemoglobin levels (29). Animal studies have shown that a HFD and overnutrition increase the incidence of diabetes in male rather than female Swiss Webster mice (30, 31). Subsequent studies further demonstrated that the highest incidence of diabetes occurred in mice with a HFD habit from birth, and changing this habit could reduce the incidence of diabetes (32). Therefore, dietary habits are crucial for the prevention and management of diabetes. It is recommended to adopt a healthy diet, including moderate consumption of low-carbohydrate and low-fat foods, and increase vegetable intake to reduce the risk of diabetes.

There have been further investigations into the mechanisms underlying the association between a HFD and diabetes. Previous studies conducted by the authors have demonstrated that short-term high-fat feeding specifically leads to hepatic fat accumulation, increased glucose uptake and metabolism in the liver, as evidenced by increased lactate production, upregulation of pyruvate carboxylase activity, and upregulation of serine synthesis (glycolytic bypass pathway), resulting in hepatic steatosis and stimulating gluconeogenesis, activating PKC-epsilon and JNK1, leading to hepatic insulin resistance (33, 34). Additionally, a HFD can cause sustained upregulation of intestinal fructose metabolism-related glucose transporter 5 (Glut5), ketohexokinase, and aldolase B. When consumed with high fructose intake, a HFD promotes fructose metabolism in the small intestine, releasing glyceric acid into circulation. Elevated levels of glyceric acid lead to pancreatic cell damage and impaired glucose tolerance, increasing the risk of diabetes (35). From an epigenetic perspective, a HFD can induce DNA methylation, histone modifications, and increased expression of non-coding RNAs, resulting in decreased transcriptional activity of key β-cell genes, leading to insulin resistance and the development of diabetes-related epigenetic mechanisms and metabolic regulation (36). The pancreatic β-cell surface expression of Glut-2 is crucial for glucose-stimulated insulin secretion. GlcNAcT-IVa glycosyltransferase is required for the residency of Glut-2 on the β-cell surface. Long-term consumption of a HFD leading to diabetes is associated with reduced expression of GlcNAcT-IV and decreased Glut-2 glycosylation occurring simultaneously with Glut-2 endocytosis (37). The HFD model in *Drosophila melanogaster* reproduces the phenotype of mammalian diabetes. Glass bottom boat (gbb) is the Drosophila homolog of mammalian transforming growth factor-beta (TGF-β), and its expression is increased under HFD conditions. Tribbles is a negative regulator of AKT and a target gene of Gbb signaling in adipose tissue. HFD-induced TGF-β/Gbb signaling leads to insulin resistance by increasing tribbles expression. Knockdown of Tribbles rescues these metabolic phenotypes, providing a critical target for the treatment of diabetes (38).

### 2.3 Dyslipidemia

Diet is considered the cornerstone of blood lipid management. High-fat foods refer to foods that are high in fat content. The authors gleaned from previous investigations that dietary intervention with a Mediterranean diet in children with primary hyperlipidemia can significantly improve the lipid profile of patients with familial hypercholesterolemia and the polygenic hypercholesterolemia subgroup, and achieve target levels of low-density lipoprotein cholesterol (LDL-C) and non-high-density lipoprotein cholesterol (HDL-C) in PH subjects (39). T2D patients adopting a Mediterranean diet showed significant reductions in postprandial plasma triglycerides, triglyceride-rich lipoproteins, and plasma remnants of cholesterol (40). In a randomized controlled trial, it was observed that a low-cholesterol diet can alleviate dyslipidemia in obese women (41). A summary of data from 12 studies involving nearly 1,500 obese patients found that a low-fat diet can reduce body weight and improve various metabolic syndrome markers such as blood lipids and insulin resistance (42). The diet of the Paleolithic era was typically similar to a low-carbohydrate diet, characterized by low fat content, which significantly reduced total cholesterol, triglycerides, and LDL-C (43). Human plasma deep lipidomics revealed that a diet rich in monounsaturated fatty acids can reduce diacylglycerols and increase triacylglycerols (44). Therefore, dietary therapy for dyslipidemia has become a hot research topic. Analysis of multiple studies through a meta-analytical approach unveiled that consumption of millet for a period of time can reduce cholesterol and triglyceride levels in the body, or help improve hyperlipidemia (45). Whole grain foods lower LDL-C and TC levels (46). Traditional dairy products increase fasting LDL-C, while dairy products with modified fatty acid composition do not increase LDL-C (47). α-linolenic acid, a major plant-derived ω-3 polyunsaturated fatty acid, has been shown to improve lipid profiles, mainly by significantly reducing triglycerides, total cholesterol, LDL-C, VLDL cholesterol, total cholesterol/HDL-C ratio, and LDL cholesterol/HDL-C ratio (48). A study in normal weight and overweight women found that drinking orange juice can prolong postprandial lipid levels (49). Another interesting study found that participants who regularly consumed shrimp had a significantly lower risk of heart failure and hyperlipidemia compared to those who did not consume shrimp, suggesting that eating more shrimp can reduce hyperlipidemia (50).

Further research has found that a HFD can affect the gut microbiota, leading to an imbalance in lipid homeostasis in the body and affecting various tissues and organs, including influencing microglial cells and the vagus nerve that regulates satiety in the brain, promoting cholesterol accumulation in the liver, disrupting lipid oxidation in muscles and energy storage in adipose tissue, affecting the integrity of the intestinal barrier, and causing dyslipidemia. Conversely, dietary changes that restore balance to the gut microbiota can improve lipid metabolism through the aforementioned mechanisms (51).

### 2.4 NAFLD

Dietary adjustments have been proven to be a highly effective approach in controlling and mitigating NAFLD (52, 53). A survey analysis of 13,000 healthy individuals revealed that a high-salt diet can predict NAFLD during a 5-year follow-up period (54). HFDs cause gut dysbiosis and increased gut permeability, which promote the development of NAFLD (55). Studies have shown that an imbalance in fatty acid metabolism, following the hepatic uptake of dietary fats, leads to the accumulation of intrahepatic triglycerides (IHTAG), which is significantly associated with NAFLD (56). In addition to saturated fats, the consumption of carbohydrate-rich foods is also a potential risk factor for fatty liver disease (57). Feeding mice a HFD for 9 weeks resulted in elevated blood cholesterol and triglyceride levels, leading to NAFLD (58). Exploring recent research, noteworthy findings indicated that a HFD can modulate the gut microbiota, affecting susceptibility to obesity-related NAFLD (59).

Improving the quality of diet, reducing the consumption of pro-inflammatory foods, and increasing the intake of anti-inflammatory foods are potential dietary interventions to reduce the risk of NAFLD (60). An 8-week dietary intervention in boys (11–16 years old) with NAFLD effectively alleviated pediatric fatty liver and improved liver cell damage (61). In a randomized controlled trial, both Mediterranean diet and low-fat diet interventions improved hepatic steatosis in NAFLD patients after 12 weeks (62). Another study found that a green Mediterranean diet (which includes additional intake of Mankai and green tea, while limiting red and processed meat consumption) further reduced intrahepatic fat content and significantly lowered the incidence of NAFLD (63). Intermittent fasting, by maintaining circadian rhythm, inducing weight loss, improving cardiac metabolic parameters (such as blood pressure, cholesterol and triglyceride levels, insulin and glucose metabolism), reducing inflammatory markers, endoplasmic reticulum stress, oxidative stress, autophagy, and endothelial dysfunction, and modulating the gut microbiota, can improve non-alcoholic steatohepatitis (64, 65). In adolescents with NAFLD, a low-sugar diet not only reduces liver fat and alanine aminotransferase (ALT) levels but also decreases hepatic *de novo* lipogenesis (DNL) and fasting insulin (66). Targeting liver mitochondria with drugs has been shown to improve NAFLD in non-human primate models of metabolic disorders (67). The authors gleaned from previous investigations that NAFLD patients have increased levels of blood bile acids, altered primary and secondary bile acid ratios, and impaired bile acid signaling pathways mediated by FXR and FGF4 in the liver (68). In addition, alleviating HFD-induced NAFLD in rats can be achieved by inhibiting the Akt/mTOR/ULK1 pathway to activate lipophagy in the liver (69). Furthermore, FGF21 has been found to be closely associated with the occurrence and progression of NAFLD and may serve as a key therapeutic target for NAFLD (70, 71).

### 2.5 Hypertension

The consumption of a Western diet, characterized by its high fat content, low fiber intake, or excessive salt levels, has been found to suppress beneficial gut microbiota, provoke pro-inflammatory immune responses, and elevate blood pressure (72, 73). In studies involving parent-offspring genetic relationships, it has been found that a HFD in mothers increases blood pressure in offspring rats (74–77). Numerous studies have demonstrated that diet plays a role in regulating hypertension through its effects on the kidneys, sympathetic nervous system, and vascular system, with the gut-immune axis being a recent focus of research (72, 78, 79). A HFD can increase cardiac hypertrophy, left ventricular remodeling, impaired contractile function, and gene expression changes associated with hypertension. Animals with hypertension induced by high-fat food consumption show decreased atrial natriuretic factor mRNA, reduced myosin heavy chain isoform switching (from α to β), increased citrate synthase activity, and increased medium-chain acyl-CoA dehydrogenase activity. These effects can be reversed by a low-fat diet (80). HFD-induced disturbances in carbohydrate metabolism, endothelin-1, TGF-β, and connective tissue growth factor (CTGF) can lead to structural abnormalities in the vascular wall, dysregulation of fibronectin release, and elevated blood pressure in elderly rats (72, 81). Furthermore, a HFD can induce sensitization to hypertension through upregulation of the brain renin-angiotensin system and central pro-inflammatory cytokines, leading to the induction of Ang II (82).

Maintaining a healthy dietary habit is crucial for controlling blood pressure. A multicenter, single-blind, randomized controlled trial based on a traditional Chinese diet design, which included reducing sodium intake by half, increasing potassium intake, moderately reducing fat intake, increasing protein and carbohydrate intake, and doubling dietary fiber intake, found that this dietary pattern effectively lowered blood pressure (83). Another study in rats found that a HFD increased blood pressure, but after intervention with a calorie-restricted diet, blood pressure, body weight, and blood glucose levels decreased, and the expression of nicotinamide phosphoribosyltransferase (NAMPT), insulin receptor (IR), sirtuin 1 (SIRT1), and complex II proteins increased in liver tissue (84). These findings highlight the interrelationship between a HFD and hypertension in the body.

### 2.6 Hyperuricemia

The incidence of hyperuricemia continues to rise, and there is a significant causal relationship with dietary habits. Investigations have revealed that dietary-induced uric acid production accounts for approximately one-fifth of the total uric acid in the body (85). In addition to high-purine and high-fructose diets, long-term consumption of high-fat foods has been identified as a potential risk factor for elevated uric acid levels and the development of hyperuricemia (86–88). Animal experiments have also frequently utilized HFD to induce hyperuricemia models (89). In a study conducted by Yu et al. (90) in 2018, it was found that feeding rats a high-fat diet supplemented with 10% yeast extract for six weeks successfully induced a hyperuricemia model, accompanied by alterations in the gut microbiota, which aligns with the findings of a study by Guo et al. (91). Hsu et al. (92) observed that rats fed a high-fat diet exhibited significant increases in body weight and uric acid levels compared to those fed a normal diet. Furthermore, Sun et al. (93) discovered that long-term consumption of a high-fat diet in C57BL/6 mice led to various renal abnormalities, including proteinuria, elevated blood urea nitrogen and creatinine levels, impaired renal function, and increased apoptosis of renal tubular cells. In clinical settings, dietary interventions have shown promise in improving uric acid levels. A study involving 235 obese patients demonstrated that low-fat, Mediterranean, and low-carbohydrate diets, implemented over a 6-month weight loss period followed by an 18-month maintenance period, all resulted in significant improvements in serum urate levels (87). Similarly, research on obese patients revealed that dietary modifications combined with increased physical activity significantly ameliorated uric acid levels in the body (94).

Further in-depth research has revealed that a high-fat diet increases uric acid levels in adipose tissue and upregulates the expression of the XOR gene and the PPARγ gene, which is associated with fatty acid oxidation, while dietary interventions, such as the consumption of New Zealand spinach, have been shown to reduce plasma uric acid levels in obese individuals by potentially decreasing uric acid production and secretion through a decrease in XOR expression in adipose tissue (95). In comparison to a normal diet, a high-fat diet (HFD) exacerbates uric acid crystal deposition and fibrosis in the kidneys of mice with hyperuricemic nephropathy. This results in renal dysfunction, characterized by elevated serum creatinine levels, renal fibrosis, and tubular injury scores. Additionally, HFD significantly increases the levels of NLRP3, ASC, caspase-1, and IL-1β (96). Moreover, HFD can induce structural disruption and cellular degeneration in the liver and pancreas, leading to increased plasma, pancreatic, and hepatic uric acid levels (97). On the other hand, the Mediterranean diet and the DASH diet have been found to regulate purine metabolism in the host, thereby lowering serum uric acid levels and preventing hyperuricemia. The mechanisms underlying this effect may involve the modulation of urate transporters, inhibition of XO activity sites, suppression of the TLR4/NF-κB signaling pathway, and modulation of the NLRP3 signaling pathway (98, 99).

## 3 HFD can disrupt the balance of the gut microbiota in the body

Dietary interventions can modulate the balance of the gut microbiota in the body. HFD has been shown to suppress beneficial bacteria and promote the growth of detrimental bacteria (Table 1). Short-term consumption of a high-fat diet in mice has been found to induce obesity-related changes in the gut microbiota, which can be partially reversed upon cessation of the high-fat diet. This suggests that diet-induced dysbiosis has a certain degree of structural resilience, highlighting the potential of dietary interventions in the treatment of metabolic disorders caused by dysbiosis (101, 109–111). This has also been supported by numerous animal and clinical studies. A re-analysis of sequencing data from 27 studies investigating the relationship between diet and gut microbiota revealed consistent effects of HFD on the gut microbiota structure, including an increased Firmicutes/Bacteroidetes ratio and an increase in the relative abundance of *Lachnospiraceae, Ruminococcaceae*, and *S24-7 Muribaculaceae* (10). *Lachnospiraceae, Ruminococcaceae, and Muribaculaceae* enrichment may suggest intricate interactions within the gut microbiota in the context of HFD-induced metabolic diseases. The observed microbial changes align with previous research on inflammatory bowel diseases, implying a compelling link between high-fat diets, gut microbiota, and metabolic disorders (112). It is reasonable to speculate that gut inflammation plays a pivotal role in the intricate relationship among HFD, gut microbiota, and metabolic diseases. A study on gene knockout mice found that a high-fat diet disrupts the circadian rhythm of both mammalian and gut microbial communities, leading to a decrease in the relative abundance of *Lactobacillaceae* and an increase in the relative abundance of *Peptostreptococcaceae* and *Clostridiaceae*, regardless of genotype (12). Furthermore, studies in rats have shown that both high-fat and high-fat/high-sugar diets can alter the abundance of gut microbiota, resulting in a decrease in the abundance of *Bacteroides/Prevotella* spp.*, Clostridium cluster IV, Methanobrevibacter* spp.*, Alistipes, and Muribaculaceae,* and an increase in the abundance of *Enterobacteriaceae, Blautia, and Akkermansia*. It has also been found that these high-abundance bacteria are positively correlated with inflammatory metabolites such as arachidonic acid, stearic acid, and dihydroxy-sulfate (104).

**Table 1:** Effects of high fat diet on gut microorganisms and body.

No.	Specimen type	Specimen Source	Technology	Microbial composition alteration	Metabolites and other alterations	First author, year
1	C57BL/6NRj mice	Small intestine and caecum	16S rRNA	*Akkermansia muciniphila* ↓	The intestinal mucosa of mice exhibits lipid droplet accumulation, along with decreased levels of Mucin-2 protein and reduced expression of Mucin-2, −3, −4, and − 13 mRNA in the intestine.	Koshiro Sonomoto (2016) (100)
2	C57BL/6 J mice	Fecal samples	16S rRNA	*Bifidobacterium* and *Akkermansia***↓** *Dorea* ↑	Not listed	Sandra Infante Villamil (2018) (101)
3	C57BL/6 mice	Distal ileum luminal contents	16S rRNA	*Lactobacillaceae***↓,** *Peptostreptococcaceae* and *Clostridiaceae* ↑	A high-fat diet has a greater impact on shaping the composition of the gut microbiota than the deficiency of the Reg3γ gene.	Katya Frazier (2023) (12)
4	Sprague–Dawley rats	Fecal samples	16S rRNA	*Bacteroides/Prevotella* spp., *Clostridium cluster IV, Methanobrevibacter* spp.**↓** *Enterobacteriaceae*↑	Serum levels of TNF-α and IL-6, as well as leptin, significantly increased, while MCP-1 levels also increased. Moreover, gene RNA levels of PPARMA-1 1cv 4mmoemand MORF-1 in muscle were elevated.	Kelsey H. Collins (2016) (102)
5	C57BL/6 J mice	Fecal samples	16S rRNA	*Blautia* and *Akkermansia*↑ *Alistipes* and *Muribaculaceae***↓**	Increasing production of 2-oleoylglycerol	Ming Yang (2023) (103)
6	Adult Balb/c male mice	Fecal samples	16S rRNA	*Ruminococcaceae, Deferribacteres*, and *Verrucomicrobia, Bacteroides*, *Akkermansia, Alitipes,* and *Mucispirillum* ↑	Elevated levels of pro-inflammatory metabolites, including arachidonic acid, stearic acid, and 2,3-dihydroxypropyl sulfate.	Rong Tan (2021) (104)
7	C57BL/6 J mice	Intestinal contents (cecum and colon)	16S rRNA	*Desulfovibrionaceae*, *Rikenellaceae RC9 gut group*, and *Mucispirillum*,↑ *Lactobacillus*, *Bifidobacterium*, *Akkermansia*, *Faecalibaculum*, and *Blautia*.**↓**	Increased expression of genes involved in non-absorptive carbohydrate metabolism pathways, as well as the potential for intestinal pathogens and inflammation.	Botao Wang (2020) (105)
8	Sprague–Dawley rats	Fecal samples	16S rRNA	Firmicutes and Bacteroidetes.↑ *Bifidobacteria*, and, *Lactobacillus***↓**	Increased adipocyte size, decreased protein abundance of UCP-1, PPAR-α, PGC1-α, and Tbx-1 in white adipose tissue, and increased liver ROS levels, oxidative proteins, and GSSG/GSH ratio.	Azalia Avila-Nava (2016) (106)
9	C57BL/6 J mice	Fecal samples	Whole-genome shotgun sequencing	*Bacteroides,* *Oscillobacteria, Desulfovibrio,* and *Proteus* ↑	HFD upregulated gut microbial functions associated with oxidative-reduction reactions, leading to increased levels of ROS. This resulted in the disruption of intestinal barrier, induction of mitochondrial dysfunction, and apoptosis of intestinal epithelial cells.	Nianyi Zeng (2023) (107)
10	C57BL/6 mice	Fecal samples	16S rRNA	*Bacteroides, Parabacteroides, Alloprevotella,* and *Ruminococcus***↓** *Akkermansia muciniphila, Desulfovibrionaceae* and *Bilophila* ↑	Decreased gut SCFA levels and increased LPS levels.	Tiande Zou (2022) (108)

↑: Increased; ↓: Decreased.

By improving dietary habits, it is possible to ameliorate HFD-induced dysbiosis and reduce the abundance of bacteria that produce endotoxins, such as through increased gut SCFA levels and decreased circulating lipopolysaccharide (LPS) levels (108). The Mexican diet, characterized by low fat content, has been found to lower liver reactive oxygen species (ROS) levels, oxidative proteins, and the GSSG/GSH ratio, while increasing the abundance of *Bifidobacterium.* This diet also improves HFD-induced dysbiosis and cognitive impairment (106). Another interesting study found that offspring of mice exposed to HFD may have a higher risk of social dysfunction, which is mediated by changes in the gut microbiota and exhibits intergenerational effects. It was also found that these negative effects can be reversed through gut interventions (113).

Diseases caused by HFD or intestinal barrier disruption due to dysbiosis of the gut microbiota are closely related. Studies have found that HFD can stimulate pro-inflammatory signaling cascades, increase cytokines (TNF-α, IL-1β, IL-6, and IFN-γ) that disrupt the barrier, and decrease cytokines (IL-10, IL-17, and IL-22) that promote barrier formation, thereby enhancing intestinal permeability. HFD negatively regulates the composition of the intestinal mucosa and increases the abundance of species that disrupt the barrier (100, 114, 115). The team led by Hongwei Zhou first revealed the dynamic changes in the gut microbiota in a high-fat feeding model in mice. By comparing mice fed with HFD and normal diet, they found that HFD immediately altered the composition of the gut microbiota and subsequently damaged the integrity of the intestinal barrier. HFD upregulated gut microbial functions associated with oxidative-reduction reactions, increased ROS levels, disrupted the intestinal barrier, induced mitochondrial dysfunction and intestinal epithelial cell apoptosis. The superoxide anions produced by gut bacteria contributed to intestinal barrier damage by injuring intestinal epithelial cells, exacerbating downstream diseases. This study provides a foundation for a deeper understanding of the underlying mechanisms by which dysregulation of gut microbiota function affects host health and deserves attention (107).

While the aforementioned viewpoints demonstrate the detrimental effects of a HFD on human health and the gut microbiota, there are also differing opinions due to the complexity and diversity of the gut microbiota, as well as inconsistencies in research regarding dietary composition and experimental animal strains. Some argue that the changes in gut microbiota induced by HFD have uncertainties and require further investigation (115). A recent study has once again highlighted the complexity and uncertainty of the impact of diet on the body. Wang et al. (105) found that increasing the consumption of high-fat foods can enhance the diversity of the gut microbiota, increase the production of SCFA, and promote energy expenditure, contrary to previously accepted views. Researchers conducted a comparative analysis between mice fed a HFD and those fed a low-fat diet (low-fat diet, or simply low-fat), examining the composition and dietary intake of both diets. They discovered that the high-energy-density HFD reduced food intake, resulting in significantly higher fiber intake in mice fed HFD compared to those on a low-fat diet, as HFD contained a higher fiber content (105). Furthermore, mice fed HFD consumed more fiber than their low-fat diet counterparts, leading to an increase in SCFA concentrations in the gut, which was associated with an increase in the abundance of specific bacteria in the HFD group (105). This study provides valuable insights and new directions for researchers to consider when assessing the effects of dietary formulations and whether differences in specific nutrient intake may interfere with the outcomes of animal experiments.

## 4 Dysbiosis of the gut microbiota can lead to metabolic disorders

With the advancement of germ-free mouse technology and the integration of advanced techniques such as single-cell sequencing, metagenomics, metabolomics, and artificial intelligence, we are now able to comprehensively and extensively study the relationship between the gut microbiota and diseases. This enables us to provide more precise methods and strategies for the prevention, diagnosis, and treatment of diseases.

### 4.1 Obesity

With the gradual application of metagenomic sequencing technology in the study of gut microbiota, it has been discovered that there is a close relationship between gut microbiota and obesity, and gut microbiota can serve as a biomarker for the prevention and treatment of obesity. The earliest evidence of the involvement of gut microbiota in the development of obesity comes from a comparison between germ-free (GF) mice and conventionally raised mice. A series of groundbreaking studies by the Gordon team found that although GF mice had a higher food intake, they were still leaner than mice with a normal gut microbiota (116). Furthermore, even when fed a high-fat, high-sugar diet or a diet rich in animal fats, GF mice did not become obese (117, 118). All of these findings demonstrate the close relationship between gut microbiota and obesity.

Early research on gut microbiota mainly focused on its role as a biomarker for diagnosing obesity. However, as research progressed, it was found that gut microbiota and its metabolites can act as signaling molecules and participate in the regulation of obesity. Gut microbiota can influence the efficiency of energy acquisition from digested nutrients and fat storage in the body. By transferring fecal microbiota transplantation to the host or feeding probiotics, obesity phenotype indicators can be improved, and the levels of HDL-C, LDL-C, adiponectin, leptin, and TNF-α can be regulated (116, 119–121). Animal experiments have also found that obese mice receiving microbiota from post-bariatric surgery mice showed enrichment of mucin-degrading *Akkermansia muciniphila* or *Bacteroides fragilis*, increased levels of tryptophan-derived metabolites, SCFAs, and acylcarnitines, increased brown fat mass and activity, increased energy expenditure, and improved immune homeostasis in white adipose tissue (122). Clinical studies have also observed similar phenomena, and the mechanism behind this may be that the gastric bypass-shaped gut microbiota reactivates the energy expenditure of metabolically active brown adipose tissue through the bile acid receptors FXR-TGR5, thereby reducing obesity in diet-induced obese patients (121).

Scientists have identified several gut microbiota that have a promoting or exacerbating effect on obesity. In a study involving Duroc pigs, it was observed that when *Prevotella copri* from these pigs was introduced into germ-free mice, it triggered chronic inflammatory responses in the mice via the TLR4 and mTOR signaling pathways (123). This colonization significantly upregulates the expression of genes related to fat formation and accumulation, while inhibiting the expression of genes related to fat breakdown, fat transport, and muscle growth (123). Furthermore, the increased abundance of *Prevotella copri* is significantly correlated with elevated concentrations of obesity-related metabolic products (LPS, branched-chain amino acids, aromatic amino acids, and arachidonic acid metabolites) in pig serum (123). Another study in BALB/c mice found that supplementation with the probiotic *Bifidobacterium breve* downregulates the expression of fatty acid translocase and fatty acid binding protein 1, and inhibits the transport and uptake of fatty acids into the liver, thereby reducing hepatic triglyceride levels (124). Groundbreaking research has also revealed the significant role of *Akkermansia muciniphila* in obesity. *Akkermansia muciniphila* improves glucose tolerance in mice, upregulates insulin sensitivity, and reduces the expression of liver damage markers, thereby inhibiting fat accumulation and ultimately delaying the development of type 1 diabetes and obesity (125, 126). Clinical randomized double-blind controlled trials have also demonstrated the effectiveness of *Akkermansia muciniphila* in weight loss (127). These results, which include evidence from comparisons between germ-free and conventionally raised mice, as well as subsequent investigations into the impact of gut microbiota on energy acquisition, fat storage, and the modulation of obesity-related markers, collectively underscore the pivotal role of gut microbiota in the development and regulation of obesity. Therefore, it is evident that manipulating gut microbiota composition holds significant promise as a strategy for addressing and managing obesity.

### 4.2 Diabetes

The gut microbiota and its metabolites play a crucial role in regulating glucose metabolism. Imbalances in the gut microbiota can affect glucose metabolism by acting on various signaling molecules in the host, thereby promoting the progression of diabetes (128, 129). In a study where immunostimulatory gram-negative bacteria from the gut of obese individuals were isolated and cultured in germ-free mice, the cultured germ-free mice exhibited higher levels of the gut metabolic product lipopolysaccharide (LPS) and developed insulin resistance compared to germ-free mice exposed to the same conditions. This suggests that specific microbial communities can disrupt glucose metabolism (130, 131).

The gut microbiota is closely related to type 1 diabetes. In a mouse model, it was found that increasing the abundance of the genus *Bifidobacterium* in the gut can enhance intestinal barrier function and reduce bacterial translocation, thereby enhancing the blood glucose-lowering effect of adipose-derived stem cells (132). Subsequent studies found that transplanting the gut microbiota from drug-improved type 1 diabetic mice can increase the abundance of *Lactobacillus* and improve the reproductive ability of diabetic mice (133). A clinical trial also identified a causal relationship between an increased relative abundance of *Bifidobacterium* and an increased risk of type 1 diabetes (134). In a non-obese diabetic (NOD) mouse model, it was found that the expression of intestinal antimicrobial peptides was reduced in newborn NOD mice, leading to dysbiosis of the colonic microbiota, which induced colonic immune system imbalance and promoted pancreatic autoimmune response and the development of diabetes in adult NOD mice (135). Another study found that transplanting *Akkermansia muciniphila* into high-risk NOD mice can promote mucus production, increase the expression of the antimicrobial peptide Reg3γ, inhibit the growth of segmented filamentous bacteria, reduce serum endotoxin levels, decrease the expression of TLR in the islets, promote regulatory immunity, and delay the development of diabetes (136). Further clinical research found that daily supplementation of *Akkermansia muciniphila* significantly reduced insulin levels and insulin resistance, and the effect was better with dead *Akkermansia muciniphila* than with live bacteria (127). *Akkermansia muciniphila* may be a key bacterium in inhibiting type 1 diabetes in mice.

There has also been in-depth research on the relationship between gut microbiota and type 2 diabetes (T2DM). In Japanese adults, it was found that the abundance of *Blautia wexlerae* is negatively correlated with the risk of obesity and T2DM. Supplementation of *Blautia wexlerae* in mice can alleviate the diabetes-related weight and inflammation phenotype induced by a high-fat diet (137). Another study on Finnish adults identified four bacterial species that are significantly positively associated with the risk of T2DM: *Clostridium citroniae*, *Clostridium bolteae*, *Tyzzerella nexilis*, and *Enterococcus gallinarum* (138). In a study of Dutch adults, it was found that high abundance of butyrate-producing bacteria, including *Clostridiaceae 1, Lachnospiraceae, C sensu stricto 1, Intestinibacter*, and *Romboutsia*, is associated with a lower risk of T2DM (139). By colonizing the gut microbiota or supplementing with probiotics, insulin resistance, body mass index, and gut microbiota composition in T2DM patients can be significantly improved (140, 141). A randomized, double-blind, placebo-controlled trial found that a combination probiotic, Probio-X (composed of *Lactobacillus casei LCZ, Lactobacillus plantarum P-8, Lactobacillus rhamnosus Probio-M9, Lactobacillus paracasei Probio-M8*, and *Lactobacillus paracasei V9*), can enhance the therapeutic effect of metformin in T2DM by promoting the production of short-chain fatty acids and the bile acid pathway (142). Adding the gut commensal bacterium *Alistipes onderdonkii* to diet-induced obese mice can also reduce hyperglycemia, alleviate inflammation and glucose intolerance induced by high fat (143). Currently, it has been found that the genera *Bifidobacterium, Prevotella, Ruminococcus, Akkermansia,* and *Roseburia* are negatively correlated with T2DM, while the genera *Enterococcus, Clostridium*, and *Lautropia* are positively correlated with T2DM (144). The gut microbiota and its metabolites participate in the regulation of T2DM metabolism through the modulation of inflammatory factors, gut permeability, glucose metabolism, fatty acid oxidation, synthesis and energy metabolism, as well as multi-bacterial cooperation (144, 145). In wild-type and germ-free mice with Myd88 gene knockout, it was also found that the gut microbiota regulates liver genes through Myd88, inducing the expression of liver fatty acid-binding protein (Lbp) and impairing glucose tolerance (146). An interesting study also found that changes in gut microbiota caused by maternal gestational glucose metabolism abnormalities can affect the gut microbiota of offspring through vertical transmission. This vertical transmission can be blocked by cesarean section and cross-fostering, improving offspring glucose metabolism (147).

In conclusion, the above studies have revealed new mechanisms by which the gut microbiota regulates the prevention of diabetes, providing new support for targeted interventions of diabetes through modulation of the gut microbiota.

### 4.3 Dyslipidemia

The gut microbiota plays an important role in regulating host lipid metabolism. Studies using fecal microbiota transplantation and germ-free mouse models have revealed the pathogenic role of gut microbiota in the development of hyperlipidemia (51, 148). A recent meta-analysis of 21 randomized controlled clinical trials involving nearly 1,500 participants found that probiotics can modulate the gut microbiota and improve hyperlipidemia (149). Changes in gut microbiota have been observed in patients with hypercholesterolemia (without the use of cholesterol-lowering drugs), including depletion of *Bifidobacterium*, expansion of *Clostridium cluster XIVa*, and an increased ratio of Firmicutes/Bacteroidetes. Furthermore, antibiotic treatment in ApoE KO mouse models rapidly leads to an increase in serum cholesterol, further demonstrating the relationship between gut microbiota and blood lipids (150). In a clinical study, intervention with Lactobacillus and *Bifidobacterium* strain TMC3115 significantly reduced plasma total cholesterol and LDL-C levels in participants. The frequency of bowel movements and fecal odor were also significantly improved compared to before intervention. 16S rRNA analysis revealed that TMC3115 increased the abundance of Firmicutes, Bacteroides, and Actinobacteria, while decreasing the abundance of Proteobacteria and Clostridium. Furthermore, the ratio of serum triglycerides to *Bacteroides* and *Bacteroides* abundance were negatively correlated, and the relative abundance of Dialister was negatively correlated with serum LDL-C and total cholesterol levels (151). In studies on hyperlipidemic rats, it has been found that *Bacteroides vulgatus* improves dyslipidemia and systemic inflammation in hyperlipidemic rats induced by a high-fat diet. This may be related to changes in bile acid metabolism, short-chain fatty acid synthesis, and serum metabolomic characteristics (152). Furthermore, research has shown that reducing the relative abundance of Lactobacillus, Bacillus, Streptococcus, and Lactococcus genera, which produce bile salt hydrolase (BSH), in humans and mice leads to a decrease in BSH activity in the ileum, resulting in a significant reduction in serum and hepatic cholesterol levels (153). Microbiota-related metabolites such as bile acids, lipopolysaccharides, and short-chain fatty acids also play a role in regulating hyperlipidemia, providing potential for targeted interventions in the gut microbiota for the treatment of hyperlipidemia or lipid disorders (51, 148, 154, 155).

### 4.4 NAFLD

NAFLD is a common liver disorder in Western countries, and non-alcoholic steatohepatitis (NASH) is the inflammatory subtype of NAFLD (156). Dysbiosis of the gut microbiota leads to intestinal inflammation and barrier dysfunction, allowing microbial products to reach the liver, triggering liver inflammation and contributing to the development of NAFLD and NASH (157–160). In a Western diet-induced NAFLD mouse model, supplementation with *Lactobacillus lactis* and *Pediococcus pentosaceus* restored liver weight/body weight ratio, NAFLD score, biochemical markers, cytokines, and intestinal tight junctions (161). Another study found that recipient mice transplanted with gut microbiota from high-fat diet-responsive donor mice exhibited susceptibility to NAFLD (59). Comparative analysis of multi-omics data from germ-free and colonized mice revealed that the gut microbiota plays a crucial role in hepatic fatty acid metabolism. The microbiota promotes fatty acid desaturation and elongation through specific metabolic enzymes and the degradation of dietary fiber precursors (162).

Numerous studies have analyzed the molecular targets of gut microbiota. In a study using germ-free mice, it was found that colonization with *Akkermansia muciniphila* and *Blautia producta*, which ferment inulin together to produce acetate, inhibited the progression of NAFLD through the activation of free fatty acid receptor 2 (FFAR2), an acetate receptor (163). Additionally, transplantation of fecal microbiota from NAFLD patients increased the accumulation and activation of B cells in the liver of recipient mice, exacerbating NASH, suggesting a pathogenic role of gut microbiota in B cell-mediated NASH (164). Furthermore, a study using two groups of germ-free mice transplanted with gut microbiota from pre- and post-dietary intervention obese children (PreM/PostM) found increased endotoxemia and systemic inflammation in PreM mice, accompanied by hepatic lipid accumulation and fatty liver. The PreM microbiota may inhibit lipid metabolism-related genes by suppressing PPARα, leading to hepatic steatosis (165). Another study showed that transplantation of cecal microbiota from methylcrotonoyl-CoA carboxylase 1 (MCC1) knockout (MCJ-KO) mice into germ-free NASH mice significantly improved liver and intestinal injury, alleviating NASH progression (166). In a study involving an obese patient cohort, analysis of microbiota metagenomics, blood and urine metabolomics, and liver transcriptomics revealed a positive correlation between serum ferritin and liver fat accumulation, low gut microbiota abundance, composition, and function. Fecal microbiota transplantation from “high ferritin” donor microbiota resulted in changes in iron metabolism and fatty acid accumulation-related genes in recipient mice (167).

Metabolites produced by the gut microbiota also play an important role in the development of diseases. Liping Zhao et al. isolated three pathobionts, including *Escherichia fergusonii* B29, which overgrew in the intestines of obese patients with fatty liver disease. Colonization of germ-free mice with these bacteria induced NAFLD, while colonization with commensal bacteria, such as *Bacteroides ovatus*, which had lower endotoxin activity, did not cause NAFLD. Knockout of the waaG gene in the bacterial endotoxin synthesis pathway and knockout of TLR4 in mice both prevented inflammation and NAFLD induced by *E. fergusonii* B29, suggesting that the LPS-TLR4 interaction is the most upstream and important molecular event in NAFLD (168). Furthermore, studies have delved into the pathogenic process of gut microbiota, suggesting that gut microbiota reaches the liver through the portal vein and activates pattern recognition receptors, indirectly or directly inducing hepatocyte damage. Gut-derived bile acid signaling, lipopolysaccharide delivery, and GLP-1 affect the progression of NAFLD by regulating insulin resistance, macrophage recruitment, and adipocyte dysfunction (169). Another interesting prospective study (including 100 individuals from a weight loss surgery cohort) found increased abundance of Lactobacillaceae and Streptococcaceae in the NASH group. Lactobacillaceae was positively correlated with postprandial peripheral blood ethanol concentration, suggesting that lactobacilli may influence the pathogenesis of NAFLD through ethanol production (170). In clinical research, the gut microbiota has been used to predict advanced fibrosis in NAFLD, and the diagnostic accuracy of the models is high (171–173). These studies demonstrate that the gut microbiota and its metabolites may serve as diagnostic biomarkers.

### 4.5 Hypertension

There is increasing evidence supporting the role of gut microbiota in the development and maintenance of hypertension. However, the evidence regarding the use of fecal microbiota transplantation, antibiotics, or probiotics to modulate the microbiota for managing hypertension is still very limited (174, 175). Dysbiosis of the gut microbiota may trigger an inflammatory state by affecting the differentiation and activity of immune cells, contributing to the development of experimental hypertension and potentially playing a role in the pathogenesis of clinical hypertension. A Western diet high in fat, low in fiber, or high in salt can suppress beneficial gut symbiotic bacteria and activate pro-inflammatory immune responses, leading to an increase in blood pressure (72, 176, 177).

A study found that high salt intake can lead to gut microbiota dysbiosis in mice, particularly a reduction in lactobacilli. Subsequent experiments showed that feeding mice with *lactobacilli* could regulate TH17 cells, preventing salt-induced experimental autoimmune encephalomyelitis and worsening of salt-sensitive hypertension (79). Another study compared germ-free mice with colonized mice to analyze the impact of microbiota on organ damage in hypertension. It was found that the development of target organ damage in hypertension is to some extent dependent on the colonization status of the microbiota. Germ-free mice exhibited exacerbated target organ damage in hypertension, which was more pronounced in the kidneys than in the heart. Healthy microbiota can reduce inflammation levels and alleviate target organ damage in hypertension by regulating metabolites, such as the production of anti-inflammatory short-chain fatty acids (178). Li et al. (179) discovered that the enrichment of pathogenic bacteria *Klebsiella pneumoniae* in the gut caused intestinal damage, fecal metabolome alterations, and bacterial translocation to the kidneys, which may contribute to the development of hypertension and high blood pressure. This provides new insights for exploring prevention and treatment strategies for hypertension. Chen et al. (180) analyzed the fungal communities in saliva, oral, and fecal samples from hypertensive patients and healthy controls. They found that the oral and gut environments have unique fungal ecological characteristics. The enrichment of fungi in hypertension and the ecological relationships between oral and gut fungi, as well as the associations between oral and gut bacteria and clinical parameters, suggest that the fungal microbiome may play an important role in hypertension. In a study, the G protein-coupled estrogen receptor 1 (Gper1) gene was completely deleted in Dahl salt-sensitive hypertensive rats using CRISPR/Cas9 technology, resulting in a Gper1^−/−^ rat model. Transplanting the cecal microbiota from Gper1^+/+^ hypertensive rats to Gper1^−/−^ rats eliminated the protective effect of Gper1 gene deletion on the cardiovascular system, indicating that the gut microbiota is an important factor in the regulation of blood pressure by Gper1 (181).

Other types of hypertension have also been shown to have a close relationship with the microbiota. A clinical study on gestational hypertension found that the abundance of butyrate-producing bacteria in the gut of obese individuals was negatively correlated with blood pressure and plasminogen activator inhibitor-1 levels. Early pregnancy hypertension was associated with gut microbiota composition and butyrate production (182). Pre-eclampsia (PE) is considered a pregnancy-related hypertensive disorder associated with excessive inflammatory response. A study on PE found that the gut microbiota of PE mice was dysbiotic, with a significant decrease in the abundance of *Akkermansia muciniphila*. Treatment with *Akkermansia muciniphila* significantly reduced systolic blood pressure in PE mice (183). Clinical studies have also demonstrated the relationship between gut microbiota and PE. PE patients showed decreased abundance of Firmicutes at the phylum level, increased abundance of Proteobacteria, decreased abundance of specific genera such as *Ruminococcus* and *Hafnia*, and increased abundance of genera such as *Enterococcus* and *Shigella*. Changes in gut microbiota accompanied by alterations in short-chain fatty acids were found to contribute to the development of hypertension in PE patients (184). Fecal samples from patients with pulmonary arterial hypertension (PAH), family controls, and healthy controls were subjected to 16S and shotgun metagenomic sequencing. Inflammatory markers, gut permeability, and microbial metabolites in plasma were measured. It was found that PAH patients had a pro-inflammatory gut dysbiosis, with lower circulating short-chain fatty acids and secondary bile acids promoting pulmonary vascular disease. This suggests that modulation of the gut microbiota may serve as a potential therapeutic approach for PAH (185). Systemic lupus erythematosus (SLE) patients often develop hypertension. In an SLE mouse model, alterations in the gut microbiota associated with SLE (enrichment of Bacteroides) were found to induce hypertension by increasing Th17 cell infiltration in the aorta, although they did not induce autoimmune, renal inflammation, or endotoxin-induced inflammation phenotypes (186). In hypertensive patients, a significant decrease in fragile Bacteroides YCH46 in feces and a significant increase in serum and intestinal corticosterone levels were observed. Transplantation of fecal microbiota from healthy rats significantly reduced blood pressure in hypertensive rats, while transplantation from hypertensive rats significantly increased blood pressure in healthy rats (187–189). Lastly, an interesting human study analyzed the gut microbiota and serum metabolome of African American and Caucasian hypertensive patients and found significant racial differences (190). Understanding these differences can help develop hypertension therapies targeted to specific racial groups. All of these findings demonstrate the potential of targeting the microbiota for the treatment of hypertension or for exploring the mechanisms underlying hypertension.

### 4.6 Hyperuricemia

Hyperuricemia is a metabolic disorder caused by abnormal uric acid metabolism. Metabolites derived from gut microbiota can promote uric acid excretion, while gut transport proteins can mediate uric acid excretion. Imbalance of gut microbiota can increase gut permeability, elevate circulating levels of lipopolysaccharides, and promote chronic inflammation, leading to hyperuricemia (191). Treatment with fecal microbiota transplantation further supports this notion. It has been found that when feces from hyperuricemic mice were transplanted into normal mice, the latter showed an increase in uric acid levels (192). Chu (193) found that compared to healthy controls, gout patients had increased abundance of *Prevotella, Clostridium*, and *Bacteroides* in their feces, while *Enterobacteriaceae* and butyrate-producing bacteria were decreased. This suggests that gut dysbiosis is associated with dysregulation of urate degradation and systemic inflammation in gout and may serve as a non-invasive diagnostic marker. In a study conducted on rural residents in China (referred to as the “Discovery Cohort”), it was found that compared to individuals with normal uric acid levels, patients with hyperuricemia had decreased richness and diversity of gut microbiota, altered composition of gut microbiota, decreased relative abundance of *Ruminococcus*, and changes in gut microbiota richness, diversity, and lower relative abundance of *Ruminococcus* were also associated with high serum uric acid levels. This was validated in another study conducted on urban residents in China (referred to as the “Validation Cohort”), suggesting that gut microbiota dysbiosis may be involved in regulating serum uric acid levels (194). In a clinical trial involving acute and recurrent gout patients, fecal microbiota transplantation therapy significantly reduced serum uric acid levels, decreased the frequency and duration of acute gout attacks, and improved intestinal barrier function (195). Probiotics can alleviate hyperuricemia by absorbing purines, restoring imbalanced gut microbiota, and inhibiting xanthine oxidase (XO) activity (98). Wu et al. (196) isolated a fermented food “Jiangshui” and obtained a fermented strain called *Limosilactobacillus fermentum JL-3*. After 15 days of oral administration of JL-3 in hyperuricemic mice, there was a significant improvement in inflammation and oxidative stress markers associated with hyperuricemia (IL-1β, MDA, CRE, BUN), and serum uric acid levels were significantly lower than those in the control group. Further investigation revealed that JL-3 may degrade uric acid in the gut, improve bowel movements, regulate gut microbiota, and thereby reduce uric acid levels in the body, making it a potential adjunct therapy for hyperuricemia. Zhao et al. (197) isolated *Lacticaseibacillus rhamnosus Fmb14* from Chinese yogurt. Oral administration of Fmb14 significantly increased the abundance of Prevotella in chronic purine-induced hyperuricemic (CPH) mice and decreased the abundance of *Ruminococcus* and *Bacteroides*. This may prevent CPH in mice by regulating purine metabolism and uric acid excretion systems, and Fmb14 could serve as a novel marker and regulatory target for CPH. Additionally, Previous studies conducted by the authors have demonstrated that oral administration of plant-derived *Lactobacillus* can significantly reduce blood uric acid levels and alleviate inflammatory infiltration in liver tissues in hyperuricemic mice (198, 199).

Human uricase is a pseudogene, meaning it lacks functionality and became inactivated early in human evolution. A recent study published in Cell revealed that abundant anaerobic bacteria in the gut microbiota, such as *Escherichia coli* and *Enterococcus faecalis*, have the ability to metabolize uric acid into allantoin or lactate, as well as SCFAs, acetate, and butyrate (200). This metabolic pathway in gut bacteria compensates for the host’s lack of uricase and a conserved gene cluster involved in uric acid degradation was identified. The anaerobic uric acid metabolism by gut bacteria plays a crucial role in maintaining lower blood uric acid levels and reducing the risk of gout (197). In summary, this study reveals a novel mechanism by which gut bacteria promote uric acid homeostasis in the host, highlighting the potential significance of targeting the gut microbiota as a therapeutic approach for hyperuricemia. These findings suggest that improving gut microbiota composition through the use of probiotics or targeting beneficial bacterial metabolites may be an effective strategy for preventing or treating hyperuricemia (Table 2).

**Table 2:** Gut microbial action on metabolic diseases mechanism.

No.	Types of metabolic diseases	Gut microbes	Molecules	Obesity promoter or suppressor	Mechanism and other alterations	First author, year
1	Obesity	*Lactobacillus salivarius AP-32*; *Lactobacillus rhamnosus bv-77*; *Bififidobacterium animalis CP-9*	Not listed	Suppressor	BMI is associated with decreased levels of serum HDL-C, LDL-C, leptin, and TNF-α.	An-Chyi Chen (2022) (121)
2	Obesity	*Ackermannia mucophi*l and *Brautella*	Tryptophan metabolites and SCFAs	Suppressor	BAT mass and activity increase, leading to increased energy expenditure and improved immune homeostasis in white adipose tissue. Additionally, there is an increase in the levels of tryptophan metabolites, SCFAs, and acylcarnitine content.	Jitender Yadav (2023) (122)
3	Obesity	the gastric bypass-shaped gut microbiota	FXR-TGR5	Suppressor	Reactivating the metabolic activity of brown adipose tissue to increase energy expenditure can help reduce obesity in diet-induced obese patients.	Julia Münzker (2022) (201)
4	Obesity	*Prevotella copri*	The TLR4 and mTOR signaling pathways	Promoter	Activation of chronic inflammatory response in mice leads to a significant upregulation of genes associated with adipogenesis and fat accumulation, while simultaneously inhibiting the expression of genes related to lipolysis, fat transport, and muscle growth.	Congying Chen (2021) (123)
5	Obesity	*Bifidobacterium breve*	Not listed	Suppressor	Downregulation of fatty acid translocase and fatty acid binding protein 1 expression inhibits the transport and uptake of fatty acids into the liver, thereby reducing hepatic triglyceride levels.	Elaine Patterson (2017) (124)
6	Obesity	*Akkermansia muciniphila*	Not listed	Suppressor	Improved intestinal barrier function enhances the ability to reduce fat accumulation, insulin resistance, and dyslipidemia.	Hubert Plovier (2017) (126)
7	Diabetes	Bifidobacterium	Mucin 2	Suppressor	Increasing the expression of mucin 2 to inhibit the translocation of gut bacteria to the pancreas, while upregulating the expression of TRPM7 to increase the thickness of the mucus layer.	Wanqi Lv (2020) (132)
8	Diabetes	*Blautia wexlerae*	GLP-1 and SCFAs	Suppressor	Reducing the expression of S100a8 in adipocytes to inhibit inflammation and adipogenesis.	Koji Hosomi (2022) (137)
9	Diabetes	*Akkermansia muciniphila*	Reg3γ	Suppressor	Promoting mucus production and increasing the expression of the antimicrobial peptide Reg3γ, which helps combat *Ruminococcus torques* from the microbiota. This leads to a reduction in serum endotoxin levels and expression of toll-like receptors in the pancreas.	Arno Hänninen (2018) (136)
10	Obesity	*Akkermansia muciniphila*	Not listed	Suppressor	Lowering insulin levels, insulin resistance, and plasma total cholesterol, while reducing white blood cell count and blood lipopolysaccharide levels.	Clara Depommier, 2019 (127)
11	Diabetes	The probiotic blend Probio-X	SCFAs	Suppressor	Lowering glycated hemoglobin levels and improving pancreatic beta-cell function index.	Ye Chen (2023) (142)
12	Obesity	*Alistipes onderdonkii*	Not listed	Suppressor	Lowering high blood glucose levels, thereby alleviating inflammation and improving glucose intolerance induced by a high-fat diet.	Zhipeng Li (2023) (143)
13	Dyslipidemia	*Lactobacillus, Bacillus, Streptococcus and Lactococcus* genera	FXR-FGF15	Suppressor	Activation of the FXR-FGF15 signaling pathway reduces intestinal BSH activity, leading to a significant decrease in serum and hepatic cholesterol levels.	Fengjie Huang (2019) (153)
14	Dyslipidemia	*Bifidobacterium bifidum TMC3115*	Not listed	Suppressor	Reducing plasma total cholesterol and LDL-C levels in subjects	Ke Wang (2019) (151)
15	Dyslipidemia	*Bacteroides vulgatus*	BAS and SCFAs	Suppressor	Improved blood lipid and systemic inflammation in high-fat diet-induced hyperlipidemic rats.	Mingchao Xum (2023) (152)
16	NAFLD	Fecal Microbiota in NAFLD Patients	MyD88	Promoter	Increased accumulation and activation of B cells in the liver of receptor mice exacerbate NASH.	Fanta Barrow (2021) (164)
17	NAFLD	“High-iron protein” donor microbiota (*Pasteurellaceae* and *Micrococcacea families*)	Serum ferritin	Promoter	Serum ferritin regulates hepatic iron metabolism-related genes and induces NAFLD through ferritin-associated microbiota.	Jordi Mayneris-Perxachs (2021) (167)
18	NAFLD	*Lactobacillus lactis* and *Pediococcus pentosaceus*	SCFAs, BAs, and indole compounds	Suppressor	By modulating the gut microbiome and metabolic environment, particularly the tryptophan pathway in the gut-liver axis, improvements in the progression of NAFLD can be achieved.	Jeong Seok Yu (2021) (161)
19	NAFLD	Gut microbiota in children with genetic obesity before dietary weight loss	PPARα	Promoter	Inhibition of PPARα to suppress lipid metabolism-related genes, leading to lipid degeneration.	Ruirui Wang (2018) (165)
20	NAFLD	*Dorea genus*	methylation-controlled J protein	Suppressor	Promoting nicotinamide adenine dinucleotide (NAD+) upregulation through the gut-liver axis to increase beneficial short-chain fatty acids and delay the progression of non-alcoholic steatohepatitis (NASH).	María Juárez-Fernández (2023) (166)
21	NAFLD	*Bacteroides acidifaciens* and *Blautia producta*	FFAR2	Suppressor	Acetate produced by the gut microbiota inhibits the progression of fatty liver disease through the hepatic FFAR2 signaling pathway.	Ryo Aoki (2021) (163)
22	NAFLD	Lactobacillaceae	Ethanol	Promoter	The family Lactobacillaceae is positively correlated with postprandial peripheral blood ethanol concentration, suggesting that lactobacilli may influence the development of NAFLD through ethanol production.	Abraham S. Meijnikman (2022) (170)
23	Hypertension	*Klebsiella pneumoniae*	Tight junction proteins	Promoter	Causing intestinal injury, alterations in fecal metabolomics, and bacterial translocation to the kidneys	Jing Li (2022) (179)
24	Hypertension	*Akkermansia muciniphila*	EGFR-PI3K-AKT signaling pathway	Suppressor	Extracellular vesicles derived from *Akkermansia muciniphila* are transferred from the gastrointestinal tract to the placenta, thereby alleviating symptoms in PE mice.	Yun Chen (2023) (183)
25	Hypertension	*The gut microbiota of hypertensive Gper1^+/+^ rats.*	Gper1	Promoter	The gut microbiota plays an important role in the regulation of blood pressure by Gper1.	Harshal Waghulde (2018) (181)
26	Hypertension	*The genus Blautia.*	IL-17	Promoter	By increasing the infiltration of Th17 cells in the aorta, it induces the development of hypertension.	Néstor de la Visitación (2021) (186)
27	Hypertension	*Lactobacillus murinus*	TH17	Suppressor	Regulating TH17 cells to prevent the exacerbation of experimental autoimmune encephalomyelitis induced by salt and salt-sensitive hypertension.	Nicola Wilck (2017) (79)
28	Hyperuricemia	*Escherichia coli* and *Enterococcus faecalis*	Xanthine and lactate, as well as SCFAs such as acetate, propionate, and butyrate, are produced by the metabolism of uric acid by anaerobic bacteria.	Suppressor	Anaerobic bacteria metabolize uric acid, converting it into xanthine or lactate, as well as SCFAs such as acetate, propionate, and butyrate. This metabolic process helps to compensate for the host’s deficiency in uricase, an enzyme responsible for uric acid metabolism.	Yuanyuan Liu (2023) (200)
29	Hyperuricemia	*Lactiplantibacillus plantarum*	Nucleosides	Suppressor	By reducing the absorption of uric acid precursors by intestinal epithelial cells, there is a decrease in the synthesis of uric acid, resulting in a lowering of blood uric acid levels. This demonstrates the ability to lower uric acid levels.	Mengfan Li (2023) (199)
30	Hyperuricemia	*Limosilactobacillus fermentum JL-3*	Uric acid in the intestines	Suppressor	By degrading uric acid in the intestines, it improves bowel movements and regulates the gut microbiota, thereby reducing the level of uric acid in the body.	Ying Wu (2021) (196)
31	Hyperuricemia	*Lacticaseibacillus rhamnosus Fmb14*	SCFAs	Suppressor	By producing short-chain fatty acids, it restores the balance of gut microbiota, enhances intestinal barrier function, and reduces inflammatory responses.	Hongyuan Zhao (2022) (197)

## 5 Targeting gut microbiota modulation for metabolic disorders induced by HFD

**Figure 1:**
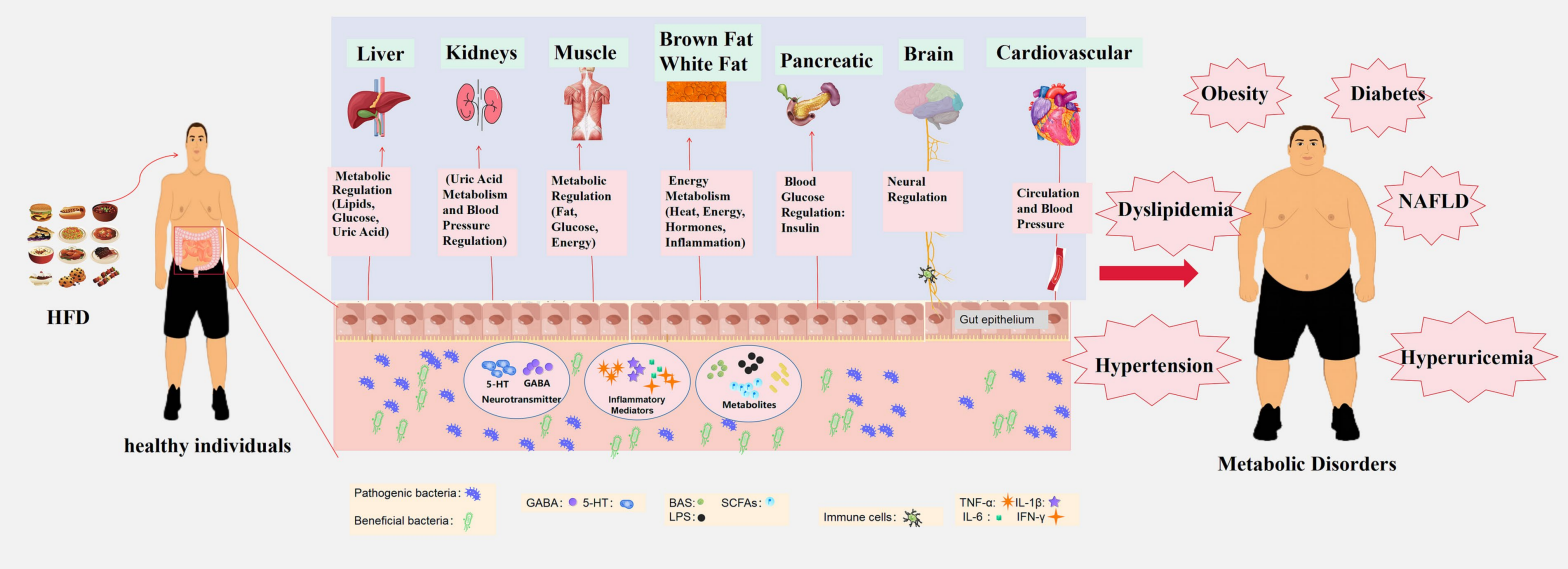
Impact of high-fat diet on metabolic disease development through gut microbiota modulation. A high-fat diet disrupts the ecological balance within the gut, reducing beneficial bacteria and increasing harmful ones. This modulation of gut microbiota leads to the production of microbial metabolites, including BAs, SCFAs, and LPS. These substances can breach the intestinal barrier and enter the circulatory system, affecting the metabolism of various organs in the body. Key organs and systems impacted by this metabolic disturbance include the liver (regulation of blood lipids, glucose, and uric acid), kidneys (blood pressure regulation and uric acid metabolism), muscles (regulation of fat and glucose metabolism), brown and white adipose tissue (energy metabolism, thermoregulation, hormonal regulation, and inflammation), pancreas (regulation of blood glucose through insulin secretion), brain (regulation of metabolism and appetite-related signals via the gut-brain axis), and cardiovascular system (influence on overall metabolism through the circulation of blood). Dysfunction in these organs and systems results in metabolic disorders such as obesity, diabetes, hypertension, dyslipidemia, NAFLD, and hyperuricemia. These interconnected metabolic disturbances are at the core of several metabolic diseases.

The Western diet, exemplified by its high-fat content, can alter the composition of the gut microbiota. The impact of this diet goes even beyond an individual’s genetic background and immune response (130, 202, 203). Changes in the composition of the gut microbiota may serve as an initiating factor for diet-induced metabolic diseases, potentially making them biomarkers (Figure 1).

### 5.1 Obesity

The gut microbiota induced by HFD can directly contribute to the development of obesity, which is a form of diet-induced obesity. Targeting the gut microbiota can improve obesity induced by HFD (204, 205). Xu et al. (206) discovered that total saponins of Panax notoginseng increased the abundance of *Akkermansia muciniphila* and *Dehalobacterium*, promoting thermogenesis in brown adipose tissue and browning of white adipose tissue in mice by activating the leptin-mediated AMPKα/STAT3 signaling pathway. This intervention helped HFD-induced obese mice resist obesity. Noëmie Daniel et al. found that supplementing yogurt in HFD-induced obese mice improved obesity by altering the gut microbiota and increasing the levels of fermentation-derived branched-chain hydroxy acids in blood and tissues (207). Hao et al. (208) observed that transplanting gut microbiota optimized with brown algal oligosaccharides increased the abundance of *Akkermansia*, reduced *Mucispirillum*, and improved liver lipid metabolism through the bile acid-retinoid acid pathway, leading to optimized blood metabolic profiles and enhanced sperm quality and fertility in HFD-induced obese male mice. Jin et al. (209) found that low-dose oral exposure to myclobutanil disrupted the gut microbiota in mice, leading to the upregulation of intestinal genes such as Fiaf and GPR41/43. This promoted dietary fat absorption, resulting in diet-induced obesity. Zhou et al. (210) discovered that mice deficient in glucuronyltransferase 2 (UGT2) had elevated bacterial enzyme 7α-hydroxysteroid dehydrogenase (7α-HSDH), which increased secondary bile acid production and excretion. This activated the intestinal FXR/FGF15 signaling pathway, inhibiting hepatic bile acid synthesis, and consequently reducing HFD-induced bile acid accumulation. Wang et al. (211) found that the gut microbiota, through intercellular signaling in immune cells, decreased the expression of long non-coding RNA gene Snhg9 in intestinal epithelial cells. This inhibited the dissociation of the CCAR2-SIRT1 complex, thereby upregulating the activity of the transcription factor PPARγ. This led to increased lipid absorption and storage, promoting host weight gain. Münzker et al. (201) discovered that post-gastric bypass surgery gut microbiota improved the obesity phenotype by regulating taurine metabolism and relying on the intestinal FXR-TGR5 signaling pathway. This reactivated brown fat thermogenesis and systemic glucose control. Intervention with *Lactobacillus gasseri* APC1472 in HFD-induced obese mice reduced body weight and fat accumulation, improved glucose tolerance, and modulated leptin and corticosterone levels while inducing changes in hypothalamic neuropeptide expression (212). Wu et al. (213) found that disrupting the intestinal HIF-2α gene lowered lactate levels in the gut, affecting the balance of gut bacteria. This, in turn, promoted thermogenesis in white adipose tissue by altering the bile acid spectrum, thereby improving host metabolism. These findings intricately elucidate the role and mechanisms of the host-genetics-gut microbiota-metabolite axis in regulating obesity, providing new insights for targeted microbiota-based therapies for HFD-induced obesity.

### 5.2 Diabetes

HFD induces systemic inflammation and exacerbates transplant rejection through both microbiota-dependent and independent mechanisms, while its role in promoting hyperglycemia is more reliant on the interplay between diet and gut microbiota. Li et al. added the commensal bacterium *Alistipes onderdonkii* to obese mice induced by HFD and found a reduction in hyperglycemia and alleviation of HFD-induced inflammation and glucose intolerance (143). Another study revealed that HFD could induce pancreatic acinar cell proliferation through gut microbiota, resulting in a 40% increase in pancreatic weight, alterations in pancreatic secretion, as reflected by decreased levels of GLP-1 and peptide YY, increased levels of glucose-dependent insulinotropic polypeptide, and changes in 32 host proteins within the gut, with pancreatic enzymes showing the most significant changes (214). Further research utilizing lipid-induced mice fed with HFD demonstrated that adding broccoli to the diet led to alterations in the diversity and structure of *Proteobacteria, Akkermansia*, and *Mucispirillum schaedleri* in the gut, improving insulin resistance (215). Sun et al. analyzed samples from Chinese Type 2 diabetes patients and found that metformin treatment significantly reduced levels of Fragile X-associated tremor syndrome bacteria, elevated levels of glycine and taurine-conjugated bile acids in the gut, and inhibited intestinal FXR signaling, ameliorating HFD-induced hyperglycemia (216). Ge′s research showed that osmanthus-derived compounds could regulate gut microbiota structure, increase SCFA content, and improve blood glucose levels, pancreatic function, and tissue inflammatory responses in Type 2 diabetes mice (217). Yan et al. found that Anemarrhena asphodeloides extract improved gut microbiota diversity, enriched beneficial bacteria such as Blautia, increased SCFA levels, and reduced potentially harmful bacteria like Klebsiella, by upregulating the expression of peroxiredoxin 4, promoting pancreatic cell regeneration and islet cell function, and improving blood glucose levels in diabetic rats (218). Daesiho-Tang (DSHT) is an anti-obesity herbal formulation, and Ahtesham Hussain et al. found that DSHT could alter the composition of gut microbiota, regulate the expression of genes related to adiponectin and resistin in adipose tissue, and exhibit anti-diabetic effects in animals fed with HFD by lowering fasting blood glucose levels (219). Molinaro et al. by analyzing liver transcriptomes of both wild-type and germ-free mice with or without myeloid differentiation primary response 88 (Myd88) gene knockout, discovered that the gut microbiota could induce liver expression of lipopolysaccharide-binding protein (LBP) gene through Myd88, consequently affecting blood glucose levels (146). In summary, HFD promotes the development of hyperglycemia through interactions with gut microbiota, involving multiple layers such as microbial diversity, pancreatic function, inflammatory responses, and gene expression. Therefore, regulation and optimization of gut microbiota may provide new therapeutic avenues for the treatment of diabetes.

### 5.3 Dyslipidemia

An HFD can induce blood lipid abnormalities through the modulation of the gut microbiota. However, targeting the gut microbiota with probiotics, prebiotics, and compounds extracted from plants can ameliorate HFD-induced blood lipid abnormalities. Han et al. utilized 16S rDNA sequencing and liquid chromatography-mass spectrometry techniques to investigate metabolic disturbances in HFD-induced metabolic syndrome in mice. They observed significant alterations in the fecal microbiota composition and glycosphingolipid biosynthesis pathways, with *g. Streptococcus* and *g. Eubacterium_coprostanoligenes* identified as key players. These bacteria were associated with changes in total cholesterol, high-density lipoprotein, and body weight, contributing to blood lipid abnormalities (220). Zhu et al. discovered that sea cucumber sulfated polysaccharides and their derivatives could inhibit dysbiosis induced by HFD, enrich beneficial *Akkermansia*, and reduce toxin-producing Proteobacteria. This led to an increase in short-chain fatty acid concentrations, a decrease in endotoxin levels, improved gut tissue indices, and reduced blood lipid levels in HFD-fed mice (221). In another study, a novel structured seaweed polysaccharide (SFP) was extracted from Ulva prolifera. SFP significantly reduced the ratio of Firmicutes to Bacteroidetes, improving gut microbiota dysbiosis in mice fed an HFD and exhibiting a remarkable lipid-lowering effect (222). Chi et al. found that Codium polysaccharides enhanced the abundance and functionality of Broutinella and Turicibacter in the gut microbiota. This led to an increase in short-chain fatty acid levels, activation of AMPK, and regulation of lipid metabolism genes such as ACOX1, ACC, and FASN, ultimately reducing blood lipid levels (223). Chen et al. conducted *in vivo* and *in vitro* experiments and discovered that prebiotic Ulva polysaccharides and lactulose oligosaccharides increased the BSH activity of *Lactobacillus rhamnosus* DM8121. This mitigated hepatic steatosis, increased cholesterol 7-α-hydroxylase expression, and BSH activity in the small intestine, thus improving blood lipid abnormalities induced by an HFD (224). CD36, a transmembrane glycoprotein, is widely distributed in the body and plays a role in lipid metabolism as a scavenger receptor. Zelei Miao et al. conducted a cohort study and found that higher n-3 polyunsaturated fatty acid (n-3 PUFA) levels in CD36 rs1527483-GG carriers were associated with improvements in blood lipids and gut microbiota characteristics (225). These findings emphasize the potential role of the gut microbiota in linking CD36 gene polymorphisms, n-3 PUFAs, and blood lipid metabolism, providing evidence for the importance of the HFD-gut microbiota-gene axis in blood lipid metabolism.

### 5.4 NAFLD

Abnormalities in the gut microbiota may promote the progression of HFD-induced NAFLD by influencing the gut-liver axis, although the precise mechanisms remain unclear (226–228). Zeng et al. systematically revealed the dynamic changes in the gut microbiota induced by high-fat feeding and found that gut bacteria produce superoxide anions, which induce damage to intestinal epithelial cells, leading to intestinal barrier dysfunction and exacerbating downstream hepatic steatosis (107). An interesting study isolated *Enterobacter cloacae B29* from obese NAFLD patients and colonized germ-free mice with these bacteria, leading to NAFLD development via the LPS-TLR4 signaling pathway (168). Another study isolated *Klebsiella pneumoniae* (Kpn) with high alcohol production (HiAlc) from the gut microbiota of NAFLD patients. They found that HiAlc Kpn, in combination with HFD, induced mitochondrial damage, disrupted the intestinal barrier, increased Th17 immune cells, and exacerbated NAFLD progression in mice (229). In a high-fat diet-induced NASH mouse model, subcutaneous injection of GLP1/2-Fc was found to modulate the enrichment of beneficial gut bacteria such as *Prevotella, Alistipes,* and *lactobacilli.* This intervention promoted intestinal epithelial regeneration, enhanced tight junctions, reduced gut permeability, and improved the gut environment (230). Yu et al. used isotope labeling to identify potential beneficial gut bacteria and their related metabolites that can directly utilize inulin. They discovered that inulin promoted the growth of *Akkermansia muciniphila*, which produces pentadecanoic acid. This, in turn, improved intestinal barrier function and protected against HFD-induced NASH in mice (231). Some studies have integrated metagenomics and metabolomics to reveal the dynamic complexity of the gut microbiota-gut microbiota-derived metabolites (such as BAs, SCFAs, etc.) axis during the treatment of NAFLD. These findings suggest that enriched gut microbiota can regulate their derivatives to alleviate HFD-induced NAFLD (232–234). Jiao et al. observed changes in the gut microbiota in NAFLD patients and HFD-induced NAFLD rats. They found increased blood bile acids, altered primary and secondary bile acid ratios, and impaired liver FXR and FGF4-mediated bile acid-related signaling pathways (68). These findings suggest that components of the FXR signaling pathway, including gut bacteria that convert primary bile acids into secondary bile acids, are potential therapeutic targets for NAFLD. María et al. induced NASH using a high-fat diet and found that enhancing mitochondrial activity reshaped the gut microbiota, promoted NAD^+^ upregulation through the gut-liver axis, increased beneficial short-chain fatty acids, and delayed NASH progression (166). Rom et al. discovered that the tripeptide DT-109, which contains glycine, reduced the significantly elevated clostridia in HFD-induced NASH mice. This intervention improved mitochondrial fatty acid oxidation, reduced liver inflammation, fibrosis, and lipotoxicity, and promoted glutathione synthesis, significantly alleviating NAFLD symptoms in experimental mice (235). Yang et al. found that *Blautia producta* in the gut and its metabolite 2-oleoylglycerol activated the GPR-119 and TAK1/NF-κB/TGF-β1 signaling pathways, inducing the activation of macrophages and hepatic stellate cells and promoting Western diet-induced NASH. These findings provide potential targets for the development of microbiota/metabolite-based NASH therapies (103). Furthermore, a study found that *Bifidobacterium pseudolongum* secretes acetate, an anti-tumor metabolite, into the portal vein, reaching the liver and binding to the liver cell G protein-coupled receptor 43 (GPR43). Activation of GPR43 inhibited the IL-6/JAK1/STAT3 signaling pathway, preventing NAFLD-HCC progression (236). In summary, the aforementioned studies provide new insights and a research foundation for a deeper understanding of the molecular associations between HFD, gut microbiota, and NAFLD, as well as potential therapies for this condition.

### 5.5 Hypertension

Research has revealed an association between a high-fat diet and hypertension, and it has also been observed that a high-fat diet can impact the composition of the gut microbiota. However, there is a relative scarcity of research on how a high-fat diet specifically targets the gut microbiota and ultimately influences hypertension. This discovery has raised a series of questions and avenues for further investigation. Hypertension is influenced by both genetic and environmental factors. In comparison to genetically predetermined factors, postnatal factors such as diet and the composition of gut microbiota may exert a greater influence on blood pressure regulation. Studies have indicated that a Western diet rich in fats, low in fiber, or high in salt can suppress beneficial gut commensal bacteria, activate pro-inflammatory immune responses, and result in elevated blood pressure (72). Conversely, a diet high in fiber, under the influence of the gut microbiota, generates short-chain fatty acids. Certain short-chain fatty acids contribute to reducing inflammatory factors, lowering blood pressure, and improving cardiac function (237–239). Furthermore, research has suggested that G-protein-coupled receptors in hypertensive patients may serve as a central link connecting diet, microbiota, and the host immune system (240–243). Previous studies have also found that a high-fat diet can regulate short-chain fatty acid metabolism in the gut microbiota. Butyric acid and propionic acid, in conjunction with GPR43 (G-protein-coupled receptor), can inhibit fatty acid breakdown, promote the secretion of the prominent fat hormone leptin, increase energy release, and suppress the synthesis of fat cells, thereby reducing fat metabolism in mice (51, 244). This indirectly demonstrates the impact of a high-fat diet on the regulation of the gut microbiota with regard to hypertension. A study conducted by Li et al. found that amlodipine (a commonly used antihypertensive drug) could lower blood pressure in a mouse model of high-fat diet-induced non-alcoholic fatty liver disease combined with hypertension by modulating the composition and function of the gut microbiota, improving intestinal barrier function, enhancing intestinal antibacterial defenses, and regulating fatty acid metabolism (245). Another study involved orally administering wasabi to rats fed a cornstarch or high-carbohydrate/high-fat diet for 8 weeks, and it was found that wasabi could regulate the gut microbiota. Hypertension was positively correlated with the high abundance of *Spirochaetes. Oscillospira* and *Butyricimonas* abundances were significantly positively correlated with systolic blood pressure (246). In summary, research into the HFD-gut microbiota-hypertension axis should focus on the metabolic products of the gut microbiota and their associated targets. This represents a novel approach to identifying diagnostic and therapeutic targets for hypertension.

### 5.6 Hyperuricemia

Currently, more research is focused on uric acid metabolism in the liver and kidneys, with only a limited number of studies addressing this process in the gastrointestinal tract (191). With our increasing understanding of the impact of gut microbiota on human health, the gut microbiome has emerged as a novel target for treatment. A randomized double-blind controlled trial found that intervention with inulin-type prebiotics resulted in enhanced uric acid degradation in feces. Moreover, the ratio of Firmicutes/Bacteroidetes in feces, which is positively correlated with uric acid degradation, increased during prebiotic intervention, leading to an improvement in serum uric acid levels in end-stage kidney disease patients (247). Another highly meaningful study involved the administration of Ferulic acid (FA) to rats on a high fructose/high fat diet (HFFD). FA significantly reduced average serum uric acid levels, blood urea nitrogen, and creatinine levels (248). The expression of urate transport proteins was reduced, while that of secretory transport proteins increased in both the kidneys and intestines of the rats. FA was also found to mitigate renal oxidative stress, inhibit the activation of the TLR4/NF-κB pathway, and suppress the activation of downstream inflammatory response markers (248). Additionally, sequencing revealed that FA altered the composition of the gut microbiota, characterized by an increase in beneficial bacteria (e.g., *Lactobacillus* and *Ruminococcus*) and a decrease in pathogenic bacteria (e.g., *Bacteroides*) (248). This study provides a foundational understanding of the relationship between a high-fat diet, gut microbiota, and hyperuricemia, and explores potential inflammatory targets. We have reason to believe that regulating uric acid metabolism in the liver and kidneys through microbiota modulation will be a major focus of future research on the impact of diet on uric acid metabolism in the body.

## 6 Limitations

This review has limitations. First, due to space constraints, it’s worth noting that metabolic diseases encompass a wide range of conditions, including but not limited to insulin resistance, metabolic syndrome, and cardiovascular diseases, in addition to the topics discussed here. Moreover, the mechanisms underlying the gut microbiota are intricate, and in real-life scenarios, the associations between high-salt diets, high-sugar diets, high-purine diets, and gut microbiota with metabolic diseases remain relevant. While this review briefly touches on these aspects, some discussions lack specific details. However, these limitations do not diminish this article’s contributions to the field of “dietary regulation and metabolic diseases.” Additionally, considering current research trends, this review primarily focuses on examining the role of HFD in metabolic diseases and the therapeutic potential of dietary regulation in this context. It also highlights the latest research developments regarding the gut microbiota’s role in treating metabolic diseases. Consequently, this review does not extensively delve into the pathological mechanisms underlying the regulation of metabolic diseases by the gut microbiota. Finally, narrative reviews have inherent limitations. To better evaluate the impact of dietary regulation via the gut microbiota on host metabolism and the effectiveness of interventions targeting the gut microbiota in metabolic diseases, future endeavors should include systematic reviews and in-depth experimental studies.

## 7 Prospect

The influence of congenital factors on human diseases is undeniable, yet recent research has revalidated the age-old adage that “disease originates in the mouth.” Diet has ascended as a paramount factor influencing metabolic diseases. The gut microbiota, akin to celestial bodies, plays an irreplaceable role in the human body’s spacetime, maintaining a close association with metabolic diseases. Dietary regimes, particularly those high in fat, can modulate the gut microbiota, fostering the development of metabolic diseases. Consequently, dietary prevention should be emphasized within the treatment paradigm. As scientific technology advances, our understanding of the relationship between diet, gut microbiota, and metabolic diseases will deepen. This research will facilitate the development of more precise preventive and therapeutic strategies, thereby enhancing the global epidemiology of metabolic diseases. It is imperative to acknowledge the uniqueness of each individual’s gut microbiota and metabolic processes. The influence of social and cultural factors on the prevalence of HFD and metabolic diseases also warrants consideration. Dietary habits and lifestyles across different regions can significantly impact the incidence of metabolic diseases. Thus, thorough research into dietary patterns within various cultural contexts is essential to devise health policies that are more attuned to local needs. Consequently, personalized dietary recommendations and treatment strategies may prove more efficacious in preventing and managing metabolic diseases. This involves employing precision medicine and genomics to customize health plans for each individual. Therefore, targeting the gut microbiota through probiotics, prebiotics, and fecal microbiota transplantation may emerge as pivotal therapeutic strategies for metabolic diseases. Although the dialog surrounding the interplay among HFD, gut microbiota, and metabolic diseases might seem “well-explored,” a notable gap persists in the literature—a thorough and in-depth exploration of the relationship among these three factors is conspicuously lacking. Prior research has predominantly concentrated on individual diseases, neglecting a holistic understanding. Hence, there is an urgent need for a comprehensive and exploratory review that penetrates this complex nexus. This paper focuses on the most recent research findings to ensure the provision of the latest insights. We also underscore emerging research methods and technologies, including germ-free mouse models, sequencing techniques, metabolomics, and microbiota transplantation, to mitigate potential confounding factors. Moreover, through this article, we strive to bridge the knowledge gap surrounding the interrelationship among HFD, gut microbiota, and metabolic diseases. Our approach transcends surface-level knowledge, probing into the intricate relationships between HFD, gut microbiota, and metabolic diseases. We explore the underlying mechanisms and illuminate the intersections between various research domains. Through this profound analysis, we aim to furnish a more comprehensive understanding of this field. Essentially, while the topics of HFD, gut microbiota, and metabolic diseases have been previously explored, this review distinguishes itself by offering the most recent insights, spotlighting cutting-edge research methodologies, and bridging critical knowledge gaps. It aspires to provide a deeper and more holistic understanding of the intricate relationships among these factors, ultimately contributing to the progression of our knowledge in this field. In conclusion, the relationship among HFD, gut microbiota, and metabolic diseases is a complex and multidimensional subject. Through profound contemplation and interdisciplinary research, we can enhance our understanding of these relationships, providing deeper insights and solutions for future healthcare. This will contribute to the improvement of prevention and treatment strategies for metabolic diseases globally, ultimately uplifting human health.

## 8 Unresolved issues

In the intricate relationship between ecological dysbiosis and metabolic diseases, the interplay between diet and gut microbiota emerges as an indispensable link. Although existing studies have attempted to unveil consistent patterns of gut microbial alterations, the diversity of the gut microbiome and its extreme sensitivity to external factors, especially diet, can lead to significant variations even among healthy individuals. Future research needs to delve deeper and more meticulously into the following key areas: (1) Interwoven Relationship of Diet, Microbiota, and Metabolic Diseases: A profound exploration of how diet influences the composition and function of the gut microbiota, subsequently affecting the development and progression of metabolic diseases. (2) Identification of Characteristic Microbes: Recognizing microbes that change in tandem with symptoms of metabolic diseases and investigating their correlation with disease severity. (3) Roles and Interactions of Pathogenic and Beneficial Strains: Identifying bacterial strains that play pivotal roles in the development of metabolic diseases and thoroughly studying their interactions with beneficial strains and potential impacts. (4) Mechanisms of Gut and Multi-organ Interactions: Elucidating the biological mechanisms between gut microbiota and metabolic diseases, focusing not only on the gut-liver axis but also on interactions between the gut and other organs such as the brain, kidneys, lungs, and heart. We anticipate that through these studies, we can establish a strain resource database related to metabolic diseases, offering robust support for future research. In this endeavor, we must acknowledge the complexity of the gut environment and consider the interactions among bacteria and between bacteria and metabolites when exploring the functions of specific strains. The intricate relationship between the microbiota and the host remains filled with unknowns. We need a deeper understanding of these interactions and how interventions targeting the microbiota affect the host. On the path to developing gut microbiota-targeted therapies related to metabolic diseases, we still face numerous challenges. However, with the continuous emergence of new technologies and methodologies, we have reasons to believe that research in this field will achieve more breakthroughs and advancements.

## Author contributions

XJ: Writing – original draft, Writing – review & editing. QC: Conceptualization, Resources, Writing – review & editing. HW: Conceptualization, Data curation, Writing – review & editing. HL: Writing – review & editing. CJ: Writing – review & editing. AG: Writing – original draft, Writing – review & editing. YZ: Conceptualization, Resources, Writing – original draft, Writing – review & editing.
